# Editorial: Th2-associated immunity in the pathogenesis of systemic lupus erythematosus and rheumatoid arthritis

**DOI:** 10.3389/fimmu.2022.975553

**Published:** 2022-07-07

**Authors:** Quanren Pan, Andrew F. Walls, Qingjun Pan

**Affiliations:** ^1^ Guangdong Provincial Key Laboratory of Autophagy and Major Chronic Non-communicable Diseases, Affiliated Hospital of Guangdong Medical University, Zhanjiang, China; ^2^ School of Clinical and Experimental Sciences, Faculty of Medicine, University of Southampton, Southampton, United Kingdom

**Keywords:** autoimmune diseases, systemic lupus erythematosus, rheumatoid arthritis, T helper cells, basophils, mesenchymal stem cells, cytokines

CD4^+^ T helper (Th) cells play a vital role in coordinating immune responses by promoting the activation and maturation of other immune cells (such as macrophages, dendritic cells, and B cells) (1). Th subsets (such as Th1 and Th2 cells) are characterized by the cytokines they secrete and their subsequent effector functions (1). Th2 cells activate and maintain humoral or antibody-mediated immune responses by producing cytokines (such as interleukin [IL]-4, IL-5, IL-6, IL-9, IL-10, IL-13, and IL-25), extracellular vesicles (EVs), and/or direct contact with target cells (2). Furthermore, Th2-associated immunity also includes other factors, such as basophils, mast cells, IgE, IgG4, Th2-related transcriptional factors (including Pparγ and Gata3), and pathways (such as Janus kinase [JAK]-signal transducer and activator of transcription [STAT] signaling and basic leucine zipper ATF-like transcription factor [Batf]/interferon regulatory factor 4 [Irf4] pathway) (2).

Increasing evidence has recently demonstrated that Th2-associated immunity targets helminths and immune responses that promote tissue repair (2) as well as plays a crucial role in autoimmune diseases such as systemic lupus erythematosus (SLE) and rheumatoid arthritis (RA), potentially contributing to disease diagnosis and prognosis (such as biomarkers), as well as targeted therapy (3–5). However, the detailed molecular regulatory mechanism of Th2-related immunity in the pathogenesis of SLE and RA and its clinical applications require further study and validation. To this end, we collected several manuscripts that analyzed the role of Th2-associated immunity in the pathogenesis of autoimmune diseases and discussed interventions targeting the relevant mechanisms.

The role of Th2-associated immunity in SLE pathogenesis is the focus of the review by Ko et al. This review summarizes lupus patients and mouse model studies on Th2-related immunity and outlines the influencing factors in the SLE microenvironment. First, IL-33 and auto-IgE activate plasmacytoid dendritic cells (pDCs) and basophils in patients with SLE. Subsequently, these activated basophils migrate to secondary lymphoid organs (SLO) and promote T cell differentiation into Th2 and Tfh2 cells. Tfh2 cells further induce B cells to differentiate into plasma cells, which produce IgE autoantibodies that consequently activate pDCs and basophils. Simultaneously, IgE autoantibodies in circulation also led to an immune complex deposition in the kidney, leading to lupus nephritis.

Notably, a study by Pellefigues et al. demonstrated that AMG853 (a bi-specific antagonist of prostaglandin D2 receptor [PTGDR]-1 and PTGDR-2) administration ameliorated lupus in Lyn-deficient female mice, whereas inhibition of PTGDR-1 or PTGDR-2 alone was ineffective. Mechanistically, AMG853 may improve lupus by inhibiting basophil activation and their subsequent recruitment to secondary lymphoid organs (SLOs), inhibiting plasmablast proliferation and autoantibody formation. However, the efficacy of AMG853 in other lupus-prone mouse models still need to be further investigated.

Sylvester et al. reviewed the concepts of autoinflammation and type 2 immunity as well as their interactions. Additionally, the authors discussed the epidemiology of a few monogenic and complex autoinflammatory diseases and the mechanisms of the interaction between autoinflammation and type 2 immunity. Delineating these mechanisms could help treat patients with various autoimmune and allergic diseases.

Recently, a study by Haddadi et al. reported that cutaneous lesions in mouse models of cutaneous lupus erythematosus (CLE) were triggered by Th2 cells, which converted to a Th1-like phenotype in response to a TLR7-driven immune environment. In this model, persistent self-reactive T-resident memory cells could serve as potential therapeutic targets.

In another study, Schubert et al. collected urine samples (56 days, 12 h intervals) from a middle-aged woman with mild SLE disease activity and measured urinary IL-6, creatinine, and protein/creatinine levels. They observed that an increase in urinary IL-6 concentration preceded an increase in urinary protein levels, which coincided with an increase in oral ulcers. While this study points to real-world clinical feedback between cytokine production and SLE symptoms, the mechanism remains unclear. In addition, owing to the heterogeneity of SLE, the delayed effect between cytokine production and SLE symptoms may not be generalized.

A review by Deng et al. focused on the dual immunomodulatory role of IL-17E (IL-25) during the progression of various autoimmune diseases. IL-25 may act as an inflammatory cytokine that promotes the production of Th2-type cytokines, including IL-4, IL-5, and IL-13, thus exacerbating allergic inflammation. IL-25 also aggravates psoriasis and Sjögren syndrome by activating innate immune cells and producing other inflammatory cytokines. In contrast, IL-25 can produce Th2-type cytokines to inhibit Th1 or Th17 differentiation, hence playing a role in RA, multiple sclerosis, and SLE. Since IL-25 plays a role in different diseases and inflammation, defining the function of IL-25 will help in its targeting for the treatment of inflammatory diseases in the future.

A review by Qin et al. showed that regulatory eosinophils (rEos) have a pro-inflammatory resolving role in RA. rEos continue to persist in the synovium of RA patients in remission and proliferate in response to innate lymphocyte (ILC)-derived IL-5 stimulation. rEos ameliorate arthritis by secreting resolvins and promoting the switch of synovial macrophages to the anti-inflammatory M2 phenotype. The authors suggest that these pro-inflammatory resolving effects of rEos could contribute to developing new therapeutic options for RA.

The role of long non-coding RNAs (lncRNAs) in SLE and RA is summarized in a review by Wu et al. In SLE, lncRNAs such as nuclear paraspeckle assembly transcript 1 (NEAT1) and growth arrest-specific 5 (GAS5) are dysregulated and hence, may be used as novel biomarkers and therapeutic targets. In RA, many validated lncRNAs, such as HOX antisense intergenic RNA (HOTAIR) and GAS5, have been identified as promising novel biomarkers for diagnosis and treatment. LncRNAs shared by SLE and RA, such as GAS5, may play critical roles in pathogenesis through diverse protein kinase pathways.

In the past decade, many studies have shown that mesenchymal stem cell (MSC) transplantation, a promising treatment option for SLE, can effectively ameliorate disease in patients with active and refractory SLE (6, 7). However, few studies have demonstrated that MSC therapy is ineffective. A review by Li et al. summarized the potential reasons for the poor effect of MSC treatment, including defects in bone marrow (BM)-MSCs in patients with SLE, factors influencing MSC proliferation *in vitro*, and the complex microenvironment of patients with SLE. The authors also proposed various MSC modification methods that may be beneficial for enhancing the immunosuppression of MSC in SLE. However, the therapeutic effects and potential adverse reactions of MSC modification in patients with SLE must be confirmed by further experimental and clinical evidence.

Further, gut microbiota dysregulation reportedly plays a vital role in the pathogenesis of SLE (8). Pan et al. elaborated on gut microbiota dysregulation in patients with lupus and mice. The authors also analyzed the mechanisms of gut microbiota dysregulation in SLE from multiple perspectives, such as molecular mimicry, gut-specific pathogenic bacterial infection, gender bias, and intestinal epithelial cell autophagy. The authors additionally proposed treatment options that may be applied to target gut microbiota dysregulation, such as oral antibiotic therapy, fecal microbiota transplantation, regulation of intestinal epithelial cell autophagy, MSC therapy, and vaccination. Thus, targeting intestinal bacteria may also be a promising strategy for SLE treatment.

Finally, a review by Chen et al. summarized mouse models of the humanized immune system based on immunodeficient mice, which better mimic the onset and progression of human disease compared to ordinary animal models. Furthermore, the authors discuss the hurdles that need to be overcome in humanized mouse models of SLE, including the short life span of mice, resulting in an insufficient observation period.

## Conclusions

This Research Topic highlights the vital role of Th2-related immunity in the pathogenesis of autoimmune diseases, such as SLE and RA. This knowledge will create the foundation for developing new therapeutic insights for Th2-related autoimmune diseases.

## Author contributions

Authors on the list have contributed a substantial amount, directly and intellectually to the work, and given their consent to publish.

## Conflict of interest

The authors declare that the research was conducted in the absence of any commercial or financial relationships that could be construed as a potential conflict of interest.

## Publisher’s note

All claims expressed in this article are solely those of the authors and do not necessarily represent those of their affiliated organizations, or those of the publisher, the editors and the reviewers. Any product that may be evaluated in this article, or claim that may be made by its manufacturer, is not guaranteed or endorsed by the publisher.

## References

[1] Marks BR Nowyhed HN Choi JY Poholek AC Odegard JM Flavell RA. Thymic self-reactivity selects natural interleukin 17-producing T cells that can regulate peripheral inflammation. Nat Immunol (2009) 10:1125–32. doi: 10.1038/ni.1783 PMC2751862 19734905

[2] Walker JA McKenzie ANJ. T(H)2 cell development and function. Nat Rev Immunol (2018) 18:121–33. doi: 10.1038/nri.2017.118 29082915

[3] Charles N Hardwick D Daugas E Illei GG Rivera J. Basophils and the T helper 2 environment can promote the development of lupus nephritis. Nat Med (2010) 16:701–7. doi: 10.1038/nm.2159 PMC2909583 20512127

[4] Kaveri SV Mouthon L Bayry J. Basophils and nephritis in lupus. N Engl J Med (2010) 363:1080–2. doi: 10.1056/NEJMcibr1006936 20825323

[5] Chen Z Bozec A Ramming A Schett G. Anti-inflammatory and immune-regulatory cytokines in rheumatoid arthritis. Nat Rev Rheumatol (2019) 15:9–17. doi: 10.1038/s41584-018-0109-2 30341437

[6] Wang D Zhang H Liang J Wang H Hua B Feng X. A long-term follow-up study of allogeneic mesenchymal Stem/Stromal cell transplantation in patients with drug-resistant systemic lupus erythematosus. Stem Cell Rep (2018) 10:933–41. doi: 10.1016/j.stemcr.2018.01.029 PMC5918528 29478901

[7] Yuan X Qin X Wang D Zhang Z Tang X Gao X. Mesenchymal stem cell therapy induces FLT3L and CD1c(+) dendritic cells in systemic lupus erythematosus patients. Nat Commun (2019) 10:2498. doi: 10.1038/s41467-019-10491-8 31175312 PMC6555800

[8] Manfredo Vieira S Hiltensperger M Kumar V Zegarra-Ruiz D Dehner C Khan N. Translocation of a gut pathobiont drives autoimmunity in mice and humans. Science (2018) 359:1156–61. doi: 10.1126/science.aar7201 PMC5959731 29590047

